# Civilianized safety practitioners reduce crime and disorder: Evidence from a quasiexperimental evaluation in San Francisco

**DOI:** 10.1093/pnasnexus/pgag275

**Published:** 2026-09-01

**Authors:** Forrest Stuart, Caylin Louis Moore, Patrick Janulis

**Affiliations:** Department of Sociology, Stanford University, 450 Jane Stanford Way—Building 120, Stanford, CA 94305, USA; Department of Sociology and Criminology, Pennsylvania State University, 229 Hammond Building, University Park, PA 16802, USA; Department of Medical Social Sciences, Northwestern University, 625 N. Michigan Ave—21st Floor, Chicago, IL 60611, USA

**Keywords:** civilian public safety, crime prevention, behavioral health, quasiexperimental evaluation, urban governance

## Abstract

Cities across the United States are experimenting with civilian-led alternatives to policing, yet rigorous evidence of their effectiveness remains scarce. This study evaluates a large-scale civilianized public safety program in San Francisco that deploys unarmed “safety practitioners” to high-crime intersections to provide consistent presence, de-escalation, and service referrals in place of traditional law enforcement. Using a generalized synthetic control design covering 624 intersections from 2018 to 2023, we estimate the causal impact of practitioner deployment on reported crime. We find that deployments significantly reduced total and behavioral health-related crimes, with smaller yet meaningful reductions in drug and violent crimes, and no change in property crime. These effects persisted across robustness checks comparing daytime (operating) to nighttime (nonoperating) hours, suggesting that observed declines are attributable to practitioner presence rather than secular trends. The findings indicate that civilianized safety practitioners—drawn largely from communities affected by incarceration, addiction, and homelessness—can function as capable guardians who lower public disorder without relying on coercive authority. By embedding trusted, nonpolice actors in high-need public spaces, cities may improve safety while reducing harmful police contact and expanding workforce opportunities for people historically excluded from the formal economy.

Significance statement Cities across the United States are searching for ways to improve safety without expanding police power. This study provides the first quasiexperimental evidence that civilianized “safety practitioners”—unarmed community members trained in de-escalation and service connection—can reduce crime and disorder in high-need public spaces. Evaluating a San Francisco program across more than 600 intersections, we find sizable and sustained reductions in total and behavioral health-related crimes, with no increase in serious offenses. These results demonstrate that visible, noncoercive civilian presence can promote safety through trust and human connection rather than enforcement. The findings highlight an emerging model of public safety that is both effective and restorative, advancing ongoing debates about the future of urban policing.

## Introduction

Is it possible to reduce crime and disorder without relying directly on the police? This question has become increasingly important as cities search for ways to address behavioral health crises, homelessness, substance use, and public disorder while minimizing the harms associated with coercive law enforcement. Interest in civilianized approaches to public safety accelerated following the George Floyd uprisings and calls to “defund the police” in 2020, but the underlying challenges long predate those events. Today, American cities face several unmet public safety needs that expose both the practical and normative limits of relying on police as the primary response to a wide range of social problems.

First, police departments across the United States face growing challenges related to capacity, retention, and public legitimacy. Studies conducted in the wake of the George Floyd uprisings suggest that some officers have reduced proactive enforcement activities—a phenomenon referred to as “de-policing” or “pragmatic avoidance” (1, 2). At the same time, many departments have experienced elevated rates of officer resignation and retirement. In cities such as Austin, Denver, and Seattle, excess losses of sworn personnel ranged from 7.5 to 16% (3). While these trends are not as pronounced in smaller and more rural jurisdictions (4), American cities continue to grapple with how best to address persistent public safety challenges amid changing institutional conditions.

Second, many of the problems that consume police resources—including low-level disorder, behavioral health crises, substance use, and unsheltered homelessness—are difficult to address through traditional enforcement strategies alone. Scholars have long described these responsibilities as forms of “dirty work” or “rabble management” (5)—recurring social problems that place police in the role of responding to poverty, mental illness, addiction, and public disorder. A growing body of research suggests that police contact in these contexts can produce unintended harms, particularly among vulnerable populations. Antagonistic encounters are linked to heightened psychological distress (6), worsened physical health (7–9), diminished social ties (10), reduced trust in public institutions (11–13), and increased risk of injury or death during police encounters (14, 15). One in four police killings, for example, involves an individual experiencing mental illness—a rate roughly 16 times higher than that observed in the general population (15). These challenges have fueled growing interest in alternative public safety models that rely less on coercion and more on prevention, relationship-building, and service provision.

Facing these challenges, community organizations and government agencies are developing community-based, trauma-informed programs to address crime and disorder through approaches that may be better suited to behavioral health crises, homelessness, substance use, and other forms of social instability than traditional law-enforcement responses alone.

Some of the most successful follow the community responder model, which dispatches social workers and mental health professionals—sometimes alongside police—to handle nonemergency 911 and 311 calls (14, 16). With expertise in behavioral health and social services, community responders aim to de-escalate disorderly behavior, stabilize individuals in crisis, and connect them to care. Evaluations show that these programs reduce crime and improve health outcomes. A Denver, CO, pilot program produced a 34% drop in disorder-related crime (17). In Seattle, WA, individuals assisted by community responders were 60% less likely to be arrested within 6 months, spent 41 fewer days in jail, and had 87% lower odds of prison incarceration over the following year compared with those who received a police response. They were also twice as likely to obtain shelter, 89% more likely to secure permanent housing, and 46% more likely to find formal employment (18).

A growing number of cities have adopted a related model, known as community violence intervention (CVI) (19). Here, community organizations hire former gang- and crime-involved individuals to calm the streets and “interrupt” retaliatory violence. These “credible messengers” draw on lived experience with incarceration, violence, and victimization to build trust with gunshot victims and their peers, steering them toward counseling, employment, and case management (20–22). Formal evaluations of CVI programs report mixed outcomes. Some find no effects or inconsistent ones, with positive results often concentrated in specific contexts. Recent reviews emphasize that while the evidence base remains uneven, CVI remains a vital part of a broader civilianized public safety infrastructure—especially when paired with efforts addressing structural violence (19, 23–29).

The popularity of these initiatives has spurred a third, related approach that assigns civilianized public safety practitioners a more preventive role. This model merges CVI's reliance on credible messengers with the behavioral health orientation of community responders and the environmental insights of ecological crime prevention. Routine Activities Theory (RAT) and Situational Crime Prevention highlight the role of “capable guardians,” or “eyes on the street,” in deterring crime and disorder (30–32). Traditionally, capable guardians include police officers, private security personnel, business employees, neighbors, doormen, janitors, and other individuals whose visible presence increases informal social control. Civilianized safety practitioners build on this logic but represent a distinctive form of guardianship. Rather than relying on formal authority or coercive power, they draw on lived experience, local knowledge, and sustained relationships to influence behavior and maintain order. As Marcus Felson, one of RAT's originators, writes: “An employee may form personal ties to a customer. When that customer enters the store drunk, the employee may exercise informal control as well as formal responsibility in getting him or her to leave with no harm done” (32, p. 56). Such interventions can spark a feedback loop—specifically, visible guardians signal that a place is cared for, prompting others to join informal control efforts, further reducing crime and disorder (33–38). In this sense, civilianized safety practitioners can be understood as a novel form of capable guardian—one whose influence derives not from the threat of sanction but from trust, familiarity, and repeated interaction.

Across a range of US cities—including New York, Seattle, San Francisco, and Los Angeles—civilianized safety practitioner programs are deploying new cohorts of guardians to strengthen informal control in areas marked by extreme poverty, homelessness, open-air drug markets, substance abuse, and recurring behavioral health crises (39–42). Building on the CVI model, these programs hire practitioners with lived experience in these issues, providing the empathy and credibility needed to build trust (43, 44) with would-be offenders and others in distress. Typically, teams of three to four practitioners are assigned to a small, defined area—such as a block face, intersection, transit station, or strip mall—on a semipermanent basis.

While practitioners remain anchored to specific locations, they are not static. Teams operate on foot and may respond to nearby disturbances within the program area, particularly when safety is at risk. Lacking vehicles, they rarely travel far from their posts. Their central task is to maintain a consistent, visible presence within a walkable radius, reinforcing informal social control through familiarity and routine.

During their multihour shifts, practitioners engage in visible activities that prevent and reduce crime and disorder: cleaning sidewalks and public spaces; forming relationships with residents, workers, and other regulars; de-escalating conflicts; directing substance users to safe consumption sites; reversing overdoses; providing basic resources (e.g. clothing, food, and sanitary items) and referrals to housing, treatment, and medical care; negotiating with drug dealers to relocate; escorting children, elderly, and disabled residents; assisting families in locating loved ones; and guiding visitors.

Unlike mobile crisis teams, practitioners are not dispatched to specific incidents. Instead, they serve as a standing presence in high-need spaces—defusing tensions and redirecting disorder before it triggers police involvement. Their preventive role and absence of coercive authority make them a formal, though distinct, alternative to policing. Embracing the community response model, practitioners aim to move upstream in the etiology of crime and disorder, addressing behavioral health crises and disruptions before escalation to police contact or arrest (14). Although these programs do not yet operate through a public-facing dispatch system like 911 or 311, their constant presence enables them to intercept incidents before community members feel compelled to call emergency services. The model does not replace police per se; it works to render police response unnecessary.

Though relatively new in the United States, civilianized public safety practitioner programs have shown promising results internationally, including a 28% reduction in total crime in England and Wales, a 25% reduction in fare evasion in France, a 10% reduction in contact crimes in South Africa, a 28% reduction in fare evasion in the Netherlands, and a 36% reduction in arrests in Australia (45–47). Programs have also reduced fear of crime and improved residents' feelings of safety (45).

However, existing evaluations lack credible causal inference. First, most rely on “admittedly “soft’ evidence”—qualitative interviews, descriptive data, or cross-sectional comparisons (48, 49). Second, analyses must distinguish effects on “focal crimes” (e.g. low-level, drug-related, and behavioral health incidents) from those on serious, “nonfocal” crimes. As Dee and Pyne note in their evaluation of a Denver-based responder program, reducing police involvement may also reduce perceived risks of apprehension (17, 50, 51), potentially “emboldening the bad guys” and increasing high-level offenses (35, 52, 53).

To address these concerns, the present study evaluates the crime-reduction effects of a civilianized safety practitioner program recently implemented in San Francisco, CA. Using a generalized synthetic control design, we estimate the treatment effect on reported crime and disorder. To our knowledge, this is the first causal analysis of such a program. While our focus is on estimating impacts, we also theorize mechanisms that may underlie these effects. Because behavioral change cannot be directly observed in our data, these mechanisms should be interpreted as plausible pathways rather than empirically verified channels.

### Urban Alchemy: San Francisco's civilianized public safety practitioner program

In 2019, Urban Alchemy, a San Francisco-based nonprofit, launched a pilot program deploying civilianized public safety practitioners in the Tenderloin Police District—a densely populated, 50-block area encompassing the Tenderloin, Mid-Market, and South of Market (SOMA) neighborhoods. Urban Alchemy, city officials, and local businesses selected the area based on greatest need. Adjacent to the central business district and city hall, the Tenderloin has the city's highest levels of poverty, unsheltered homelessness, mental illness, drug activity, and disorder. Although it contains only 5% of San Francisco's population, it accounts for 17% of total crime, 60% of drug crime, 30% of fatal overdoses, 49% of the city's homeless population, and 11% of mental health emergency calls. The median income of housed residents is $32,000—less than one-third of the city average—and one-third live below the poverty line. The district is surrounded by affluent neighborhoods whose residents increasingly demand city action on visible crises.

Following the 2020 George Floyd uprisings, the program rapidly expanded through a staggered rollout to 41 intersections by December 2022 (see Fig. S1). Practitioners are deployed 12 h per day (7 AM to 7 PM), 7 days per week. Working in teams of two to four per intersection, roughly 120–240 practitioners are on duty at any given time, with two overlapping shifts (morning: 7 AM–12 PM, overlap: 12–2 PM, afternoon: 2–7 PM).

Practitioners are unarmed and uniformed in black pants, black shirts, and green reflective vests bearing the organization's logo. Each team covers roughly a one-block radius centered on a designated “post.” While one or two members remain at the post, others patrol the surrounding area on foot. Practitioners typically stay at the same post for 6–12 months to build relationships and local knowledge. Supervisors, also on foot, each oversee about six teams and maintain contact via handheld radios, coordinating support for major incidents (e.g. overdoses, acute psychotic episodes, and physical conflicts) and reallocating staff when coverage gaps arise. Following such events, practitioners promptly return to their posts. Deployment across intersections remained uniform and independent of crime frequency.

Urban Alchemy publicly describes itself as a trauma-informed, civilian alternative to policing: “We believe that calling the police shouldn’t be the default answer to poverty and desperation. We can’t address trauma, addiction, and mental illness with the same approach we use to tackle crime.” Practitioners' shifts proceed in two phases. During the first hour, teams clean sidewalks and public spaces, removing debris, feces, and drug paraphernalia while conducting wellness checks on unhoused individuals and encampments. For the remainder of the shift, they build trust and prevent disruptions by greeting passersby, checking in with businesses about recent issues, and referring individuals to social services and shelters (e.g. safe consumption sites, food pantries, and welfare offices). Referrals occur through personal interaction and repeated contact: practitioners may walk individuals to nearby services, provide directions, or share information. Although service uptake is not tracked, field reports suggest these informal connections often mark the first step toward stability for people experiencing housing insecurity, addiction, or untreated behavioral health conditions (54).

Practitioners are trained and equipped with naloxone nasal spray, which they use to reverse roughly 800 overdoses per year. They reportedly conduct over 10,000 conflict de-escalations annually. Practitioners are prohibited from using physical force and are instructed not to intervene in severe violent offenses due to safety and legal constraints.

As in CVI programs, practitioners are explicitly recruited for lived experience with poverty, homelessness, substance use, and the criminal legal system. Urban Alchemy reports that 95% have been incarcerated and 92% identify as Black, Indigenous, or People of Color. Practitioners draw on these biographies and relationships to curb disorder and respond to behavioral health crises.

Unlike many community responder programs, Urban Alchemy operates independently of the police. The practitioner program is not integrated into 911 or 311 dispatch, and when officers arrive in response to calls, practitioners must yield jurisdiction—though they may provide contextual information as any civilian might. While the San Francisco Police Department (SFPD) publicly acknowledges Urban Alchemy's role in prevention, it has not altered deployment practices in the program area. Urban Alchemy offers no direct phone number for residents or businesses, but its consistent, visible presence allows locals to communicate needs quickly and directly.

### Measuring the impact of Urban Alchemy's civilianized public safety practitioner program on crime

The study analyzes the impact of the safety practitioner program on focal and nonfocal offenses using a generalized synthetic control method with a staggered treatment adoption structure (55, 56). First, we estimate the impact of the program on crime during operating hours, comparing treatment intersections before and after launch to a synthetic control of nondeployment intersections. Second, we assess differences between operating and nonoperating hours, using nonoperating periods as an additional control. Because crime patterns often differ systematically between daytime and nighttime hours, we do not assume that these periods are directly comparable in level or trend. Rather, the generalized synthetic control framework constructs weighted controls based on pretreatment day/night differences, allowing treatment intersections to be compared against counterfactual intersections exhibiting similar preintervention temporal patterns. As shown in Figs. 2 and S4, the synthetic control procedure closely reproduces these pretreatment dynamics, with estimated treatment effects centered near zero prior to deployment. Under this approach, identification comes from deviations from these established patterns following deployment rather than from any assumption that daytime and nighttime crime rates are equivalent. Together, these analyses yield 39,312 intersection-month observations, enabling precise estimation through temporal variation across a large panel dataset.

Crime data come from the SFPD's Incident Report Dataset (IRD), collected under the federal National Incident-Based Reporting System. The IRD includes all incidents reported by officers or the public, regardless of arrest outcome (see Table S6). Reports must be approved by a supervising sergeant or lieutenant. Citations are excluded unless an incident report was also filed (e.g. a moving violation involving firearms or narcotics). Each report includes the date, time, category, and location, anonymized to the nearest intersection. Because some local agencies (e.g. US Park Police, Bay Area Rapid Transit Police) are not required to file with SFPD, missing data are possible. These outcomes capture reported crime incidents—not arrests, citations, or enforcement activity. Our analytic focus is on changes in crime prevalence, though reductions in punitive contact may represent a meaningful secondary effect.

To prepare data, we coded each incident dichotomously as occurring within or outside the program's jurisdiction (i.e. deployment versus nondeployment intersections). Practitioners are deployed to entire block faces between intersections; thus, any intersection contiguous with a practitioner block face was classified as within program coverage. Noncontiguous intersections were coded as outside coverage. Incidents were also classified by operating hours (7 AM–7 PM) versus nonoperating hours (7:01 PM–6:59 AM).

As in most place-based evaluations, this study relies on officially recorded incident reports—the dominant and most practical measure of outcomes in hot spot research. Such data capture all recorded incidents regardless of arrest and are widely regarded as the broadest, most consistent measure of criminal events. Sherman et al. note that incident-level data provide the “widest ongoing data collection net for criminal events in the city” (57, p. 35). Similarly, Lum (58) finds that incident reports offer more accurate, stable measures than arrests or citizen-generated calls-for-service. Following this literature, we treat incident reports not as a proxy for victimization but as a standardized and replicable indicator of crime prevalence—particularly suitable for assessing interventions aimed at reducing visible disorder.

Incidents were coded into five offense categories. Focal offenses—explicitly targeted by practitioners—include drug crimes (e.g. possession and sale) and behavioral health-related crimes (e.g. disorderly conduct, mental health detention, trespassing, and drug offenses). Nonfocal offenses—untargeted by practitioners—include violent (e.g. assault, homicide, and robbery) and property (e.g. arson, burglary, and vandalism) crimes. We also examined total crime, encompassing all categories plus miscellaneous offenses (e.g. fraud and suspicious occurrence). Categories were derived from FBI Uniform Crime Reporting standards and prior studies of low-level crime, disorder, and behavioral health offenses (59–61). We could not measure unreported crime, as the IRD includes only formally reported incidents (see Tables S1–S6).

To construct a control group, we included all nondeployment intersections in SOMA, Civic Center, Tenderloin, and Union Square—geographically adjacent areas with comparable foot traffic, commerce, homelessness, and behavioral health crises. These neighborhoods share the same policing district and city policy context, making them appropriate counterfactuals. Because deployment sites may systematically differ from nondeployment sites, we used a generalized synthetic control method that weights control intersections to approximate treated ones during preintervention periods (62). This approach follows the logic of a traditional difference-in-difference analysis but provides more flexibility compared to traditional estimation techniques (e.g. two-way fixed effects), such as easily accommodating staggered roll-outs of interventions and cases where the assumption of pretreatment parallel trends is unlikely to hold, such as the current dataset (see Materials and methods for additional detail). However, given the potential for nearby intersections to be included as controls and displacement effects to impact the results, we also conducted a sensitivity analysis removing adjacent nonintervention intersections from being included in the analysis to further examine the robustness of our results.

In summary, our main analysis compares pre–post changes in focal and nonfocal crimes during operating hours between deployment intersections and their synthetic controls, then repeats the comparison to evaluate differences between operating and nonoperating hours.

## Results

Our analysis draws on monthly crime incident data spanning 63 months, from September 2018 to November 2023, capturing 12 months before and after the full rollout of the Urban Alchemy program. During this period, safety practitioners were deployed to 41 intersections across San Francisco. We compare trends in these “treatment” intersections with those in 583 control intersections that never received practitioner coverage. Using a generalized synthetic control method, we estimate effects across five crime categories. First, we assess changes in crime during operating hours between treated and synthetic control intersections. Second, we evaluate changes in daytime versus nighttime crime, leveraging nonoperating hours as an additional control. This combined approach isolates changes linked to practitioner presence during active deployment hours.

**Figure 1:**
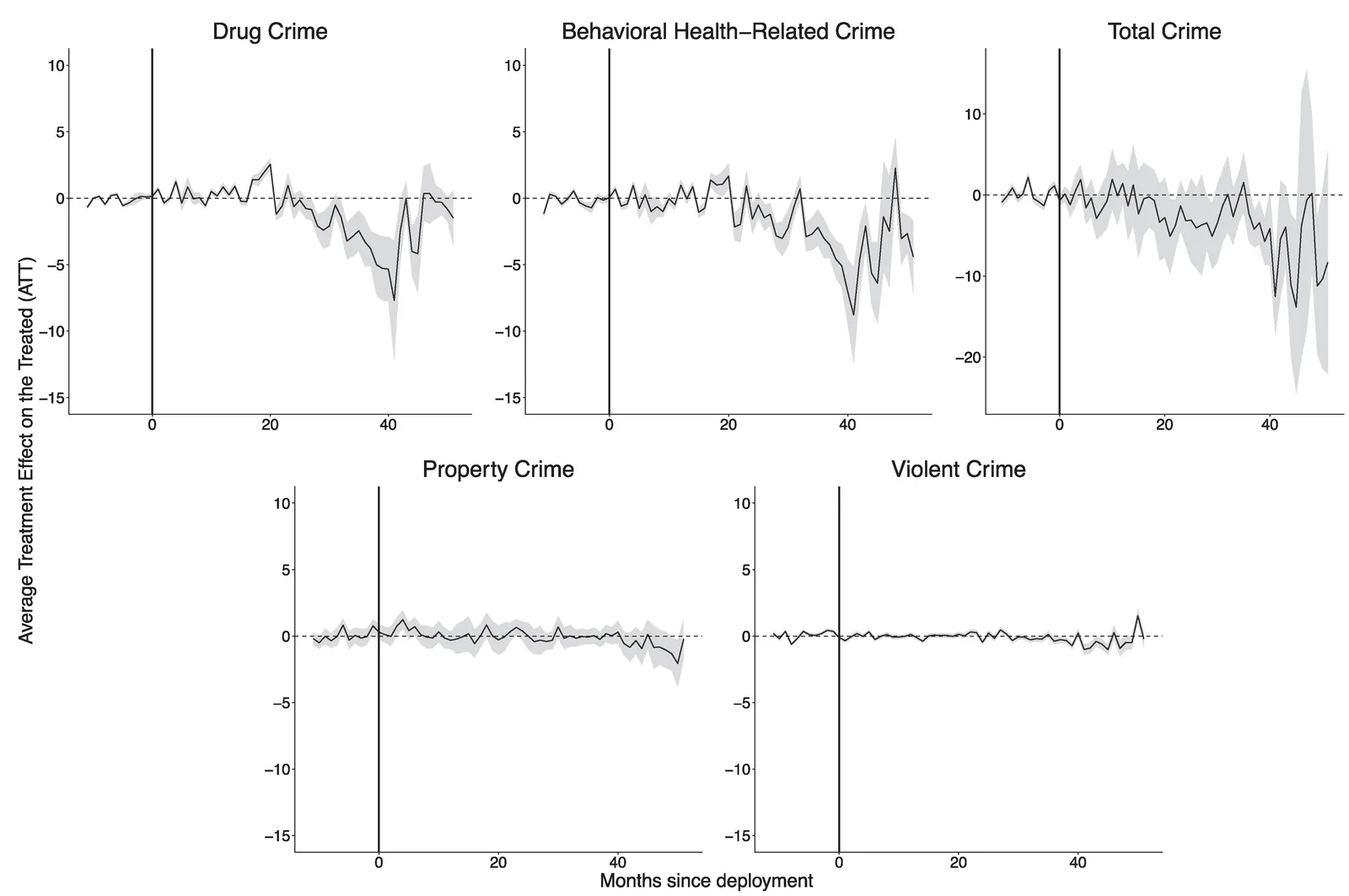
ATT for day crimes.

**Figure 2:**
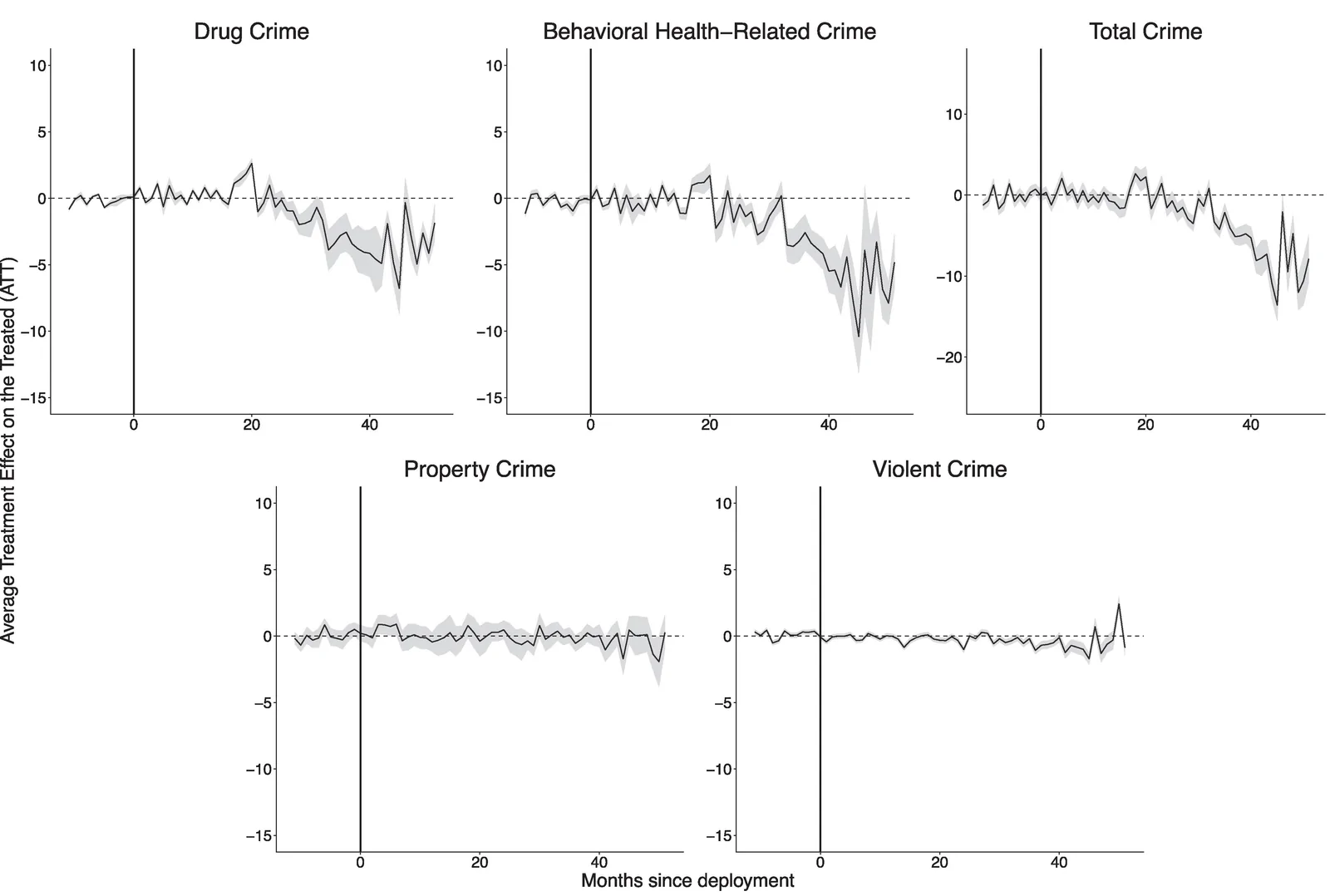
ATT for day/night crime difference.

Table 1 reports estimated treatment effects across crime categories. Results are based on two models: (i) comparing daytime crime in treated versus synthetic control intersections and (ii) comparing changes in crime during operating (7 AM–7 PM) versus nonoperating (7 PM–7 AM) hours. Our effect estimate—the average treatment effect on the treated (ATT)—represents the change in monthly crime incidents attributable to practitioner deployment, relative to a weighted synthetic control. Across both models, we find statistically significant reductions in behavioral health-related and total crime in treated areas, with drug and violent crime showing significant day/night differences. Figure 1 displays the ATT over time since deployment for all outcomes during operating hours, and Fig. 2 presents the day/night ATT comparison. Figures S3 and S4 show mean values for intervention and synthetic control sites, respectively.

**Table 1:** ATT by analysis approach and outcome.

	ATT [95% confidence interval]	*P*-value
Day crimes only		
Drug	−0.47 [−1.04, 0.10]	0.104
Behavioral health-related	**−0.90 [−1.55, −0.24]**	**0**.**007**
Total	**−1.81 [−3.41, −0.21]**	**0**.**026**
Property	0.06 [−0.36, 0.48]	0.774
Violent	−0.05 [−0.13, 0.02]	0.144
Day/night crime difference		
Drug	**−0.52 [−1.02, −0.02]**	**0**.**041**
Behavioral health-related	**−1.01 [−1.61, −0.41]**	**<0.001**
Total	**−1.09 [−1.53, −0.65]**	**<0.001**
Property	0.03 [−0.37, 0.42]	0.900
Violent	**−0.24 [−0.30, −0.18]**	**<0.001**

Bold indicates statistically significant.

### Reductions in drug crime

The intervention was associated with a statistically significant reduction in drug-related offenses in the day/night analysis but not in daytime-only models. During practitioner operating hours, treated intersections experienced a nonsignificant 0.47 fewer drug incidents per month than controls (ATT = −0.47; 95% CI: −1.04 to 0.10; *P* = 0.104). When comparing daytime to nighttime trends, the reduction became significant (ATT = −0.52; 95% CI: −1.02 to −0.02; *P* = 0.041). These results suggest a modest but measurable daytime decline in drug crime linked to practitioner presence.

### Reductions in behavioral health-related crime

We observe strong and consistent reductions in behavioral health-related incidents, including disorderly conduct and mental health detentions. During deployment hours, treated intersections saw 0.90 fewer incidents per month than controls (ATT = −0.90; 95% CI: −1.55 to −0.24; *P* = 0.007). The day/night analysis reinforces this finding: compared with nighttime hours, daytime incidents declined by 1.01 per month (ATT = −1.01; 95% CI: −1.61 to −0.41; *P* < 0.001). These reductions indicate that practitioners are particularly effective at intercepting and de-escalating behavioral health disruptions before police involvement.

### Testing for unintended consequences: effects on violent crime

Critics have argued that reducing formal law enforcement presence could embolden serious crime (17). To test this, we analyzed violent crime separately and found no evidence of unintended increases. During practitioner operating hours, treated intersections experienced −0.05 fewer violent incidents per month relative to controls, a nonsignificant change (ATT = −0.05; 95% CI: −0.13 to 0.02; *P* = 0.144). However, the day/night comparison revealed a significant 0.24 reduction in violent crimes during the day (ATT = −0.24; 95% CI: −0.30 to −0.18; *P* < 0.001). These findings suggest that civilianized safety practitioners neither enable nor increase violent crime and may modestly reduce it.

### No detectable effect on property crime

In contrast to the reductions observed in focal and violent crimes, we find no evidence that the intervention affected property crime. Estimates in both analyses were small and nonsignificant (operating hours ATT = 0.06; 95% CI: −0.36 to 0.48; *P* = 0.774; day/night ATT = 0.03; 95% CI: −0.37 to 0.42; *P* = 0.900). These null results are consistent with the program's design, as practitioners focus on behavioral crises, drug activity, and public disorder rather than theft or burglary. The lack of property crime effects helps clarify the intervention's scope and limits.

### Broad reductions in total crime

We examined total reported crime—a composite of focal and nonfocal offenses plus other categories such as fraud or suspicious activity. Practitioners' presence was associated with sizable and statistically significant declines. During operating hours, treated intersections experienced 1.81 fewer total incidents per month than controls (ATT = −1.81; 95% CI: −3.41 to −0.21; *P* = 0.026). The day/night analysis also showed a significant daytime reduction (ATT = −1.09; 95% CI: −1.53 to −0.65; *P* < 0.001). These results suggest that practitioner deployments not only reduced targeted offenses but also contributed to broader decreases in reported crime.

### Sensitivity to exclusion of adjacent intersections as controls

One concern is that crime and disorder displaced from treatment intersections may relocate to nearby nondeployment intersections. Because these adjacent intersections are also candidates for inclusion in the synthetic control pool, such displacement could artificially inflate estimated treatment effects by increasing crime in the comparison group. To assess this possibility, we conducted a sensitivity analysis that excludes all nondeployment intersections adjacent to intervention locations from the set of potential synthetic controls. This approach creates a spatial buffer around treatment areas and provides a more conservative test of whether observed effects are driven by displacement into nearby locations. We found that the removal of adjacent nonintervention intersections as potential controls largely replicated our results (see Table S7). Of note, the only major change was to the estimate of day-only total crime (ATT = −0.45; 95% CI: −2.71 to 1.26, *P* = 0.605) which may suggest this result is particularly sensitive to displacement effects. However, we also note that day/night total crime results (ATT = −1.04; 95% CI: −1.55 to −0.52, *P* < 0.001) remained nearly identical.

In sum, across offense categories, the Urban Alchemy program produced meaningful reductions in reported crime. The strongest effects appeared for drug- and behavioral health-related incidents—the intervention's primary targets. We also find modest but significant declines in violent crime, addressing concerns that nonpolice safety models might invite serious harms. Property crime was unaffected, as expected given the program's scope. Overall, the intervention generated consistent reductions in total reported crime, demonstrating the broader public safety benefits of embedding trusted, noncoercive civilian practitioners in high-need public spaces.

## Discussion

Police departments across the United States face mounting challenges related to both capacity and legitimacy. While some departments have experienced staffing shortages, early retirements, and forms of de-policing in the wake of the George Floyd uprisings (1–4, 63), a broader challenge concerns the suitability of enforcement-based responses to behavioral health crises, homelessness, substance use, and public disorder. A growing body of scholarship suggests that many of these issues are poorly addressed through traditional law-enforcement approaches and may require alternative forms of intervention rooted in care, service provision, and relationship-building (6–16, 64). Together, these developments have accelerated interest in civilianized approaches to public safety and generated new questions about whether crime and disorder can be reduced without relying primarily on coercive authority.

This study provides the first quasiexperimental evidence on the crime-reduction effects of a civilianized safety intervention in public space. Using a generalized synthetic-control design with staggered difference-in-difference and triple-difference estimators, we evaluated San Francisco's Urban Alchemy program across 41 intersections over 5 years. Practitioner presence significantly reduced behavioral health-related and total crime, with smaller declines in drug and violent offenses and no evidence of rebound or unintended effects. These findings offer strong support for the capacity of embedded, noncoercive practitioners to enhance safety in areas traditionally managed by police. More broadly, the findings provide support for a central proposition of RAT—namely, that visible and engaged capable guardians can reduce opportunities for crime. While criminological research often focuses on police, private security, or residents in this role, our results suggest that civilianized safety practitioners may serve a similar guardianship function despite lacking formal coercive authority.

Effects were strongest in total and behavioral health-related crimes, which are the categories the model targets most via de-escalation, presence, and referral. Deployment was associated with roughly a 20% reduction in total crime and a 26.7% reduction in behavioral health-related incidents across treated intersections. To illustrate magnitude, a 20% drop in total crime equates to about 74 fewer reported incidents per month at program peak. The day/night comparison similarly suggests a 29% reduction in drug crime, a 26.7% in behavioral health-related crime, and a 20% reduction in total crimes.

Although statistically significant effects for drug and violent crimes appeared only in the day/night comparison, this may reflect smaller effect sizes or secular fluctuations rather than absence of impact.

Viewed alongside credible-messenger interventions such as CeaseFire Chicago and Brooklyn's Save Our Streets—which reduced violence by 16–34% and about 20%, respectively (23, 24, 65)—Urban Alchemy's results compare favorably. While the program targets less severe offenses, it achieves reductions comparable to the most successful CVI efforts, extending the credible-messenger framework from individual outreach to spatial guardianship in public space.

Three mechanisms likely underlie these effects. First, practitioners directly prevent focal crimes by intervening early—de-escalating conflicts, supporting individuals in crisis, and stabilizing open-air drug markets. Second, they indirectly reduce incidents by offering residents, businesses, and visitors an informal channel for dispute resolution, decreasing reliance on police (14). Third, practitioners connect people to services such as shelters, treatment, and harm-reduction sites through repeated, trust-based interactions. These relationship-driven referrals address root causes of disorder—untreated illness, addiction, housing instability—substituting care for punishment. Together, these pathways help reduce harmful police contact with vulnerable populations.

The delayed emergence of treatment effects is also consistent with the intervention's underlying model. Unlike many public safety interventions that rely on deterrence or formal sanctions, Urban Alchemy practitioners build influence through repeated interactions and relationship formation. Practitioners must first become familiar with the social ecology of a block—its residents, businesses, unhoused populations, service providers, and recurring sources of conflict—before they can effectively intervene in disputes, connect individuals to services, or reshape expectations regarding behavior in public space. To the extent that practitioners reduce crime through trust, familiarity, and informal social control, effects would be expected to accumulate gradually rather than appear immediately following deployment.

Although some figures suggest modest attenuation near the end of the observation period, we are cautious about drawing strong conclusions from these patterns. Crime trends exhibit substantial month-to-month variation throughout the study period, and the generalized synthetic control framework is designed to estimate average treatment effects despite such fluctuations. Several contextual changes—including shifts in the broader service environment during the study period—may also contribute to temporal variation in effect size. At present, however, the available evidence is insufficient to distinguish between substantive attenuation and ordinary statistical fluctuation. Future research with longer follow-up periods will be necessary to determine whether treatment effects stabilize, diminish, or continue to evolve over time.

Our outcome measures, drawn from municipal crime data, capture the bureaucratic recording of incidents rather than direct behavior. This distinction is particularly important in the context of civilianized public safety interventions. Practitioners are often deployed precisely to address situations that might otherwise generate police intervention, including behavioral health crises, interpersonal conflicts, public disorder, and disputes involving businesses, residents, and unhoused individuals. As a result, reductions in reported crime may not only reflect changes in underlying behavior but also changes in how incidents are fundamentally managed and whether they ultimately result in police contact. This possibility provides support for the model's theory of change, which mitigates police-related harms by reducing the likelihood of police contact. From this perspective, reductions in incidents reported to police indicate that practitioners have successfully diverted situations that would have otherwise attracted police attention. Future research should seek to disentangle changes in underlying behavior from changes in reporting and incident resolution, ideally through the integration of survey, administrative, and ethnographic data. Even without formal coordination between Urban Alchemy and the SFPD, officers may also have informally modified enforcement practices where practitioners were active, further shaping the relationship between observed incidents and underlying behavior.

We also note that our estimates do not adjust for variation in foot traffic or population exposure, which fluctuated during the pandemic period. Although available mobility data lacked the spatial precision needed for intersection-month analysis, the day/night design mitigates this limitation by introducing a within-site temporal control. Importantly, the design does not assume that daytime and nighttime crime rates are identical or follow equivalent trends. Rather, the generalized synthetic control framework constructs weighted comparisons based on observed pretreatment day/night patterns, allowing treatment intersections to be compared against counterfactual intersections with similar temporal dynamics. As with any day/night comparison, however, the design could be biased by contemporaneous shocks that differentially affect daytime and nighttime crime in treatment versus control intersections. The staggered rollout and close pretreatment fit between treated and synthetic control units reduce, but cannot eliminate, this possibility. Results show that while control intersections saw rising daytime crime, deployment intersections remained stable or improved, strengthening causal inference. Nonetheless, we acknowledge that without detailed intersection-month-level covariates it is possible that any matching procedures may fail to provide truly comparable control intersections, thereby weakening the design.

Finally, data-mapping limitations may lead us to underestimate effects. Because San Francisco geocodes incidents to the nearest intersection, crimes occurring on adjacent nondeployment block faces may be attributed to treated sites. Future research integrating geospatial, survey, and ethnographic data could clarify how practitioner strategies—cleaning sidewalks, mediating disputes, providing resources—translate into broader collective-efficacy gains.

A central policy question is cost-effectiveness. The economic cost of a low-level crime ranges from $780 to $8,577 (66, 67). Based on our estimates, Urban Alchemy prevented roughly 890 low-level crimes per year, saving $0.7–7.7 million annually. While the program's budget was $20.5 million, a full accounting must also include reductions in serious crime and the value generated by employment. Urban Alchemy hires primarily formerly incarcerated people, providing living-wage jobs and pathways out of recidivism (42, 43). These benefits offset long-term costs while addressing harms that conventional enforcement often deepens.

In sum, the findings provide early causal evidence that fixed, visible, noncoercive practitioners can reduce crime in high-need public spaces. The model is not a substitute for all forms of policing but a promising complement, particularly in settings where behavioral health crises, homelessness, substance use, and public disorder may be more effectively addressed through civilian, relationship-based interventions. Rather than replacing police altogether, civilianized safety practitioners appear capable of addressing a distinct set of public safety challenges through trust, familiarity, and noncoercive engagement. Replication across cities and methods, including mixed-method and longitudinal designs, will clarify scalability. For policymakers, the civilianized safety practitioner model represents a practical, humane strategy to reduce disorder, strengthen community trust, and expand economic opportunity for those historically excluded from the formal economy.

## Materials and methods

For our primary analysis, this study utilized a generalized synthetic control method under a staggered treatment structure to estimate the impact of deployments on crime in total and by type (i.e. violence, property, and behavioral health-related or drug). More specifically, we leverage pre–post data on crime across treatment versus control intersections. In addition, we also leveraged the nature of daytime deployments to include a third control compared changes in daytime (i.e. 7 AM to 7 PM) versus nighttime (i.e. 7:01 PM to 6:59 AM) crimes. Finally, we also conducted a sensitivity analysis that removed adjacent nonintervention intersections from being eligible as controls to examine the robustness of our findings to potential for displacement effects (i.e. increases in crime in adjacent control intersections).

For all analyses, we utilized the generalized synthetic control method to estimate the effect of the intervention (68). This method combines and extends the traditional synthetic control method and linear fixed effect models, both methods traditionally used to estimate results in similar quasiexperimental designs. This method estimates the ATT by creating a weighted synthetic control group from control units using a latent factor approach to approximate the treatment units from pretreatment data. It is comparable to a traditional difference-in-difference approach using a two-way fixed effects model but provides additional advantages such as accommodating staggered designs and overcoming limitations of traditional approaches such as removing the assumption of “parallel trends” and should not be biased by heterogeneity in treatment effects across units (68). More specifically, with $Y_{it}$ representing the outcome for intersection *i* at time *t*, the linear factor model for $Y_{it}$ is: $Y_{it}=\delta_{it}D_{it}+X_{it}\beta +\lambda_{i}^{\prime }f_{t}+\varepsilon_{it}$. In this equation, $D_{it}$ is the treatment indicator, $\delta_{it}$ is the heterogeneous treatment effect, $X_{it}$ is a vector of observed covariates, *β* is a vector of coefficients, $\lambda_{i}^{\prime }$ represents unit-specific factor loadings, and $f_{t}$ represents time-varying coefficients (i.e. latent factors). Control group data are used to estimate coefficients and the latent factors (i.e. $f_{t}\;$) using $Y_{it}=X_{it}\beta +\lambda_{i}^{\prime }f_{t}+\varepsilon_{it}$. Then, using the estimated coefficients and latent factors, pretreatment data are used to identify factor loadings for each treatment unit using: $Y_{it}^{\prime }=X_{it}\beta +\lambda_{i}f_{t}$. Using the coefficients, factors, and factor loadings, synthetic control outcomes are estimated by $Y_{it}^{0}=X_{it}\beta +\lambda_{i}f_{t}$ and the ATT is estimated using $\;\mathrm{AT}T_{t}=\frac{1}{N_{t}}\overset{\;}{\underset{i\in T}{\sum }}\;[Y_{it}^{1}-Y_{it}^{0}]$ (62, 68). No observed covariates (i.e. $X_{it}$) were used in the current analysis. For our first analysis, $Y_{it}$ reflects the count of daytime crimes during operating hours at intersection *i* and time *t* whereas for our secondary analysis $Y_{it}$ reflects the *difference* in daytime minus nighttime crimes at intersection *i* at time *t* (i.e. $Y_{it}=\mathrm{Daytime}\_\mathrm{Crim}e_{it}-\;\mathrm{Nighttime}\_\mathrm{Crim}e_{it}$).

We note that this analysis estimates the impact of the intervention following the earliest deployment despite unique treatment histories of 7 of the 41 intervention intersections (see Fig. S2) because other approaches are not supported using the generalized synthetic control method. All analysis was conducted using the gsynth package in R (69), used a two-way additive fixed effect, and included no other covariates because unit and time invariant confounders are inherently controlled for using the generalized synthetic control method (62, 68, 69). Cross-validation and mean square prediction error were used to estimate the number of factors.

## Supplementary Material

pgag275_Supplementary_Data

## Data Availability

All code used to generate the analyses and figures reported in this study are publicly available at https://github.com/pfsj/urban_alchemy_analysis. The primary outcome data consist of incident-level crime records from the SFPD IRD, which are publicly available through the City and County of San Francisco Open Data Portal (https://data.sfgov.org) (70). These data are provided by the City in anonymized form and are subject to the City's data use policies.
