# Supplementary material for: Civilianized safety practitioners reduce crime and disorder: Evidence from a quasiexperimental evaluation in San Francisco
Source: PNAS Nexus. 2026 Sep 1;5(9):pgag275. doi: 10.1093/pnasnexus/pgag275 (PMC13531706; doi:10.1093/pnasnexus/pgag275)
Supplement: Supplementary file 1 [file pgag275_supplementary_data.pdf]

PNAS nexus

Supplementary Information for
Civilianized Safety Practitioners Reduce Crime and Disorder: Evidence from a Quasi-Experimental Evaluation in San Francisco.

Forrest Stuart,* Caylin Louis Moore, Patrick Janulis

*Corresponding author: Forrest Stuart
Emal: fstuart@stanford.edu

This PDF file includes:

Figures S1 to S4
Tables S1 to S7

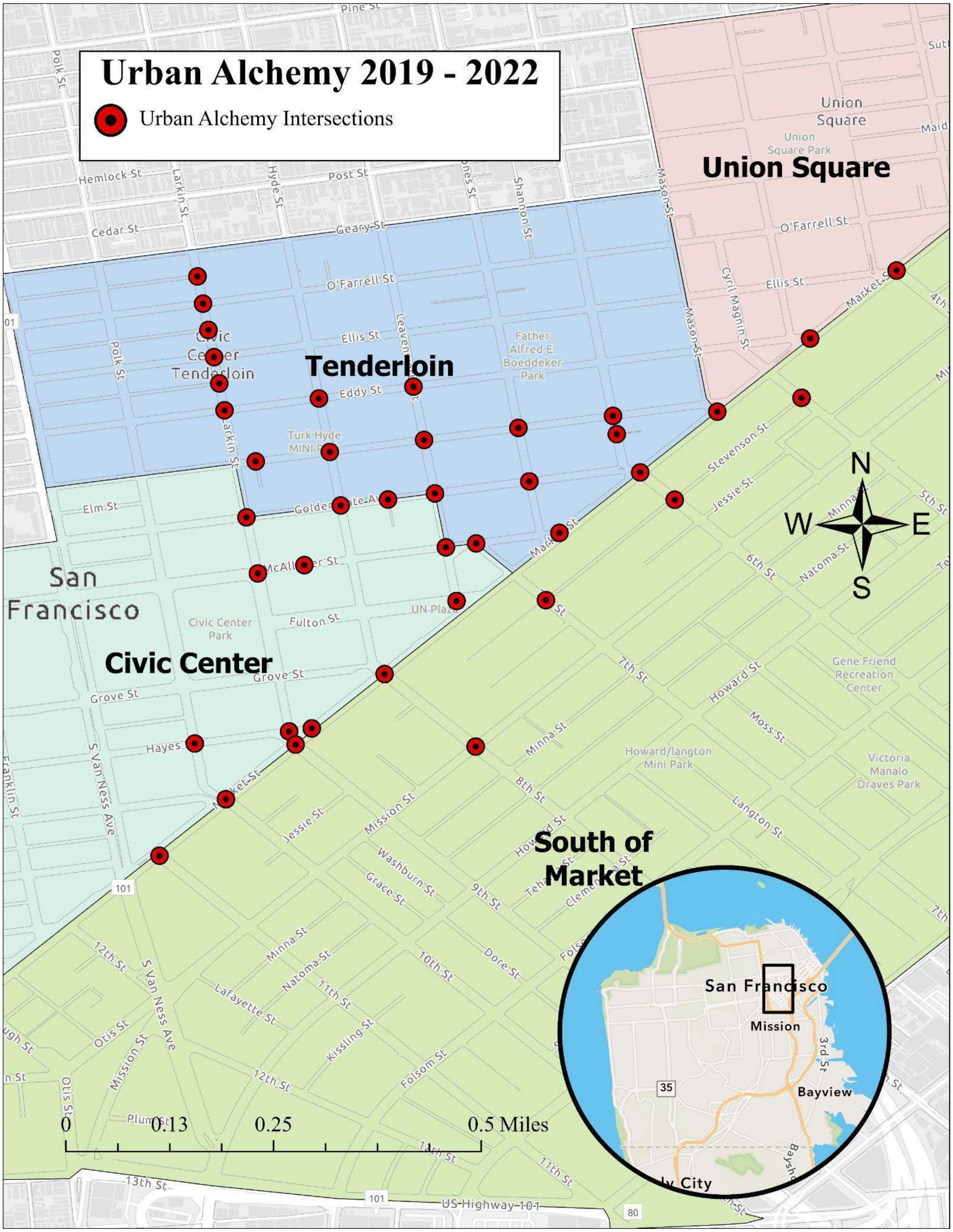

Fig. S1. Urban Alchemy Deployment 2019 to 2022. Urban Alchemy intersections are represented by black dots enclosed within red circles. The figure depicts the Tenderloin, Civic Center, South of Market, and Union Square neighborhoods of San Francisco.

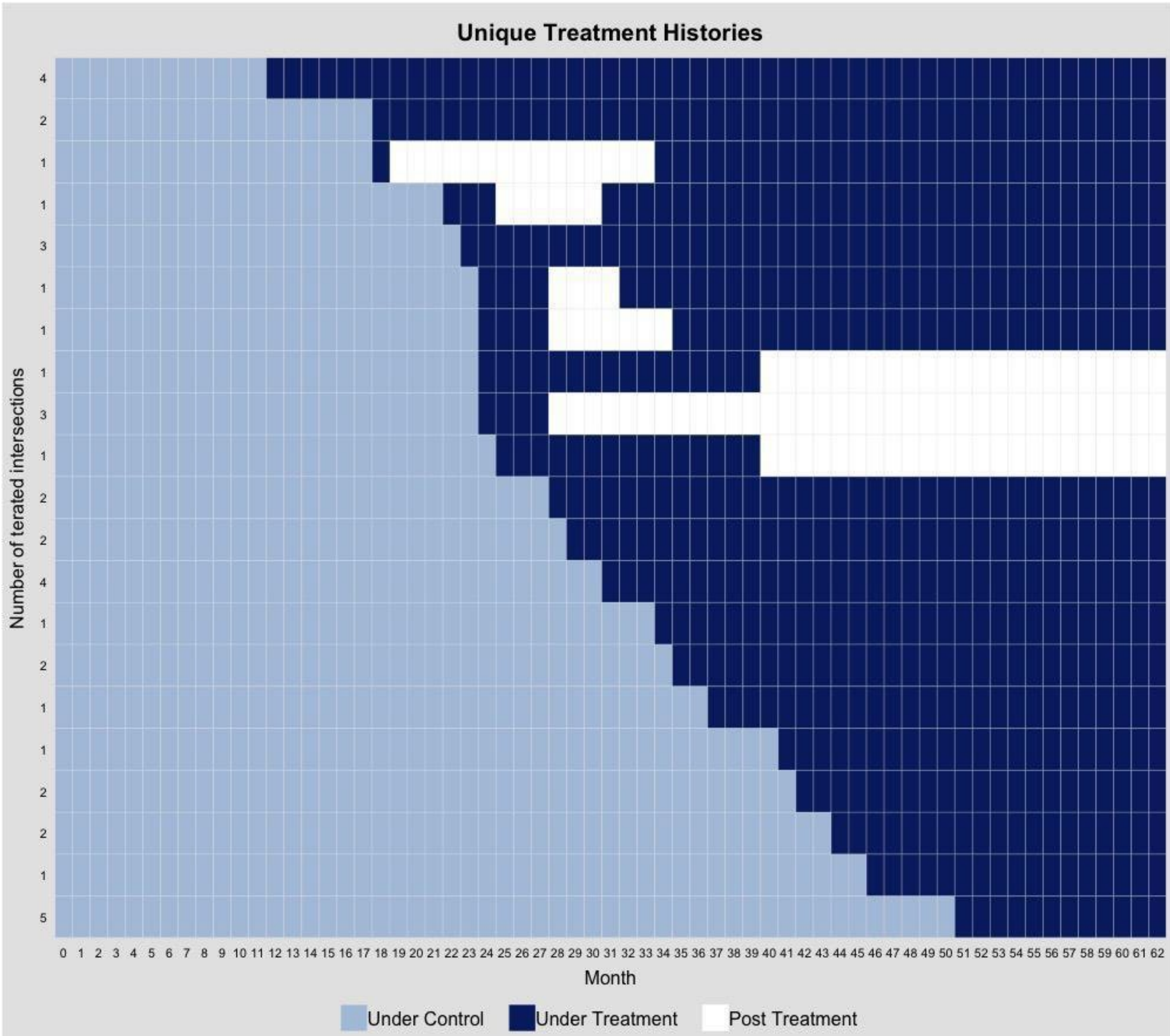

Fig. S2. Intervention Timing by Month for Treated Intersections. This figure indicates the timing of deployments by groups of intersections. The x-axis displays the time in months from the beginning of the observation period while the y-axis indicates the number of intersections that began their deployments in the given pattern.

Fig. S3. Mean values for synthetic controls and intervention for outcome in the day only analysis.

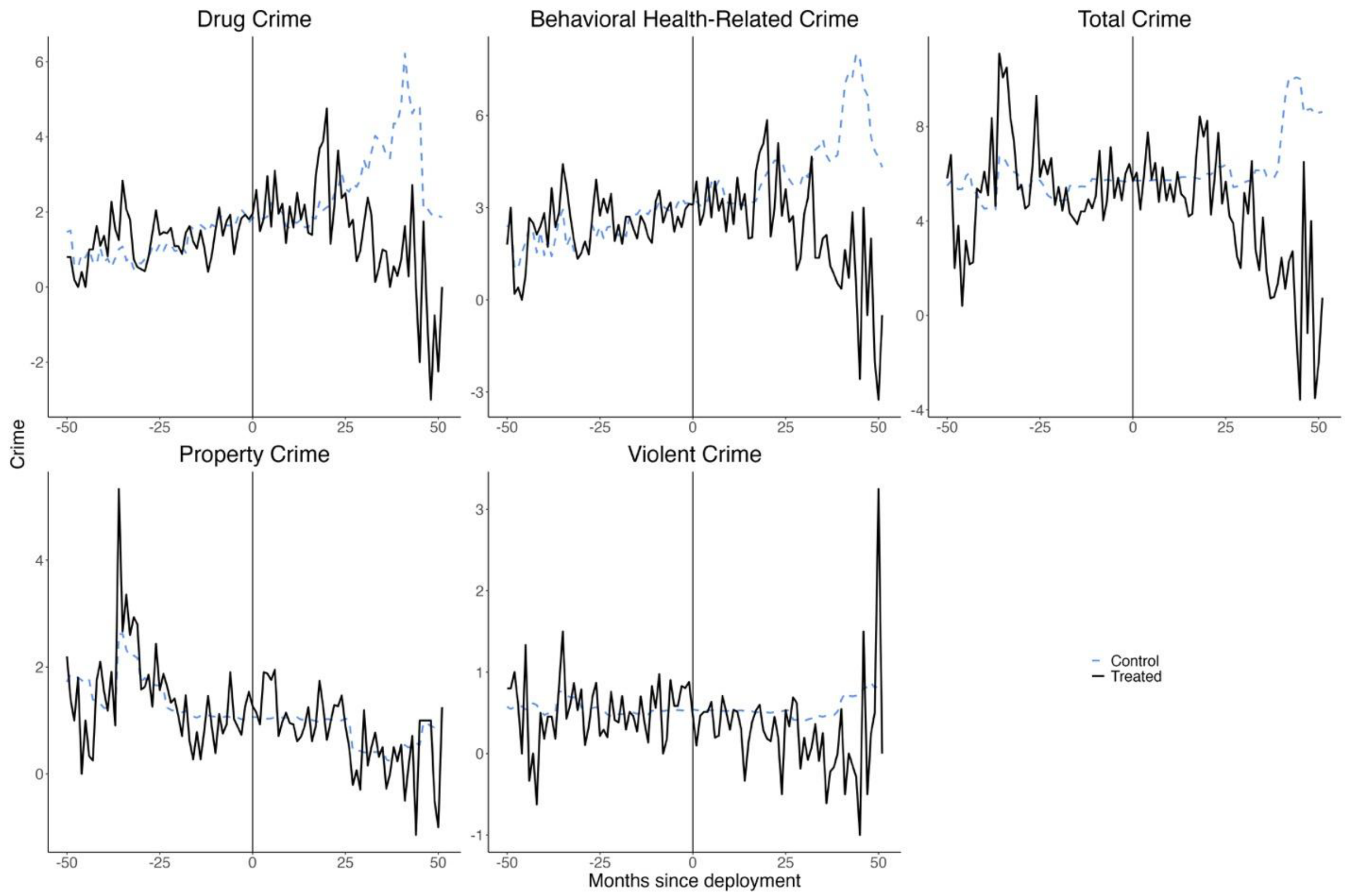

Fig. S4. Mean values for synthetic controls and intervention for outcome in the Day/Night difference analysis.

Table S1. Total Offense Type Codes.

TOTAL CRIME INCIDENTS	SAN FRANCISCO	ANALYSIS NEIGHBORHOODS
ARSON	2354	561
ASSAULT	51885	16775
BURGLARY	41764	7347
CIVIL SIDEWALKS	873	21
COURTESY REPORT	2633	439
DISORDERLY CONDUCT	15098	4463
DRUG OFFENSE	22338	16239
DRUG VIOLATION	232	105
EMBEZZLEMENT	982	338
FORGERY AND COUNTERFEITING	2265	463
FRAUD	25763	4682
GAMBLING	45	10
HOMICIDE	42	12
HUMAN TRAFFICKING (A), COMMERCIAL SEX ACTS	91	46
HUMAN TRAFFICKING (B), INVOLUNTARY SERVITUDE	3	1
HUMAN TRAFFICKING, COMMERCIAL SEX ACTS	22	5
LARCENY THEFT	230051	45736
LIQUOR LAWS	129	8
MALICIOUS MISCHIEF	57299	11895
MISCELLANEOUS INVESTIGATION	10141	1978
MOTOR VEHICLE THEFT	46703	6809
MOTOR VEHICLE THEFT?	49	14
NON-CRIMINAL	23766	7583
OFFENCES AGAINST THE FAMILY AND CHILDREN	11068	2264
OTHER	1	0
OTHER MISCELLANEOUS	46127	14789
OTHER OFFENSES	2121	755
PROSTITUTION	907	218
RAPE	216	88
ROBBERY	17623	5420
SEX OFFENSE	985	342
STOLEN PROPERTY	3106	858
SUICIDE	2	1
SUSPICIOUS OCC	17731	4414
TRAFFIC COLLISION	1	1
VANDALISM	2082	283
WARRANT	22167	10147
WEAPONS CARRYING ETC	3902	1239
WEAPONS OFFENCE	21	7
WEAPONS OFFENSE	5932	1556
TOTAL	668520	167912

Data are from San Francisco Police Department incident reports from September 2018 to November 2023, retrieved from https://data.sfgov.org. Urban Alchemy analysis neighborhoods include SOMA, Civic Center, Tenderloin, and Union Square. Any redundancies and typographical/grammatical errors are retained from the original dataset.

Table S2.
Drug Offense Type Codes.

Drug Crime Incidents	San Francisco	Analysis Neighborhoods
**Drug Offense**	22338	16239
**Drug Violation**	232	105
**Total**	22570	16344

Data are from San Francisco Police Department incident reports from September 2018 to November 2023, retrieved from https://data.sfgov.org. Urban Alchemy analysis neighborhoods include SOMA, Civic Center, Tenderloin, and Union Square. Any redundancies and typographical/grammatical errors are retained from the original dataset.

Table S3. Behavioral Health-Related Offense Type Codes.

BEHAVIORAL HEALTH-RELATED CRIME INCIDENTS	SAN FRANCISCO	ANALYSIS NEIGHBORHOODS
**CIVIL SIDEWALKS**	873	21
**DISORDERLY CONDUCT**	15098	4463
**DRUG OFFENSE**	22338	16239
**DRUG VIOLATION**	232	105
**NON-CRIMINAL**	21099	6899
**OTHER MISCELLANEOUS**	46127	14789
**OTHER OFFENSES**	2121	755
**TOTAL**	107888	43271

Data are from San Francisco Police Department incident reports from September 2018 to November 2023, retrieved from https://data.sfgov.org. Urban Alchemy analysis neighborhoods include SOMA, Civic Center, Tenderloin, and Union Square. Any redundancies and typographical/grammatical errors are retained from the original dataset.
*Other Miscellaneous includes the following: Disorderly conduct (51), Loitering (631), Other (29), for example, public transit infractions, other offenses against public justice, and trespassing (4,722).
** Other Offenses include the following: Consuming Alcohol in Public View, Municipal Code Violation, Municipal Police Code Violation, Obstruction On Streets, Sidewalks, Park Code Violation, Peddling Without a License, Permit Violation Sidewalk Sales, Aggressive Soliciting, Trash, Placing on the Street, and Other Offenses, including Loitering and Peeking into Inhabited Structure, Throwing Injurious Substance on Highway, and Throwing Object at Common Carrier, Passenger or Freight.

Table S4. Violent Offense Type Codes.

Violent Crime Incidents	San Francisco	Analysis Neighborhoods
Assault	51885	16775
Homicide	42	12
Human Trafficking (A), Commercial Sex Acts	91	46
Human Trafficking (B), Involuntary Servitude	3	1
Human Trafficking, Commercial Sex Acts	22	5
Rape	216	88
Robbery	17623	5420
Sex Offense	985	342
Total	70867	22689

Data are from San Francisco Police Department incident reports from September 2018 to November 2023, retrieved from https://data.sfgov.org. Urban Alchemy analysis neighborhoods include SOMA, Civic Center, Tenderloin, and Union Square. Any redundancies and typographical/grammatical errors are retained from the original dataset.

Table S5. Property Offense Type Codes.

PROPERTY CRIME INCIDENTS	SAN FRANCISCO	ANALYSIS NEIGHBORHOODS
**ARSON**	2354	561
**BURGLARY**	41764	7347
**LARCENY THEFT**	230051	45736
**MALICIOUS MISCHIEF**	57299	11895
**MOTOR VEHICLE THEFT**	46703	6809
**MOTOR VEHICLE THEFT?**	49	14
**STOLEN PROPERTY**	3106	858
**VANDALISM**	2082	283
**TOTAL**	383408	73503

Data are from San Francisco Police Department incident reports from September 2018 to November 2023, retrieved from https://data.sfgov.org. Urban Alchemy analysis neighborhoods include SOMA, Civic Center, Tenderloin, and Union Square. Any redundancies and typographical/grammatical errors are retained from the original dataset.

Table S6. Report Type Description Codes.

REPORT TYPE DESCRIPTION	PERCENT OF TOTAL
**INITIAL**	72.5%
**COPLOGIC INITIAL**	18.8%
**VEHICLE INITIAL**	8.7%
**TOTAL**	100%

Data are from San Francisco Police Department incident reports from September 2018 to November 2023, retrieved from https://data.sfgov.org. "Initial" and "Vehicle Initial" reports reflect police-filed incidents, which may stem from either civilian calls or officer observations, but cannot be further disaggregated. "Coplogic Initial" reports are filed online directly by civilians.

Table S7. Average Treatment Effect on the Treated (ATT) by Analysis Approach and Outcome When Excluding Adjacent Intersections.

	ATT [95% Confidence interval]	p-value
Day Crimes Only		
Drug	-0.27 [-0.76, 0.21]	0.266
Behavioral Health-Related	**-0.95 [-1.71, -0.19]**	**0.015**
Total	-0.45 [-2.71, 1.26]	0.605
Property	0.06 [-0.26, 0.36]	0.734
Violent	-0.07 [-0.14, 0.01]	0.073
Day/Night Crime Difference		
Drug	-0.45 [-1.00, -0.08]	0.096
Behavioral Health-Related	**-1.11 [-1.94, -0.30]**	**0.008**
Total	**-1.04 [-1.55, -0.52]**	**< 0.001**
Property	0.03 [-0.39, 0.45]	0.893
Violent	**-0.24 [-0.29, -0.18]**	**< 0.001**

Note: Bold indicates statistically significant.
